# Synthesis, structure, catalytic and cytotoxic activities of chlorido­(5-nitro­quinolin-8-olato-κ^2^*N*,*O*)(tri­cyclo­hexyl­phosphine-κ*P*)platinum(II)

**DOI:** 10.1107/S2056989025005766

**Published:** 2025-07-01

**Authors:** Nguyen Thi Thanh Chi, Nguyen Tran Huong Ly, Doan Duc Hieu, Luc Van Meervelt

**Affiliations:** a Department of Chemistry, Hanoi National University of Education, 136 Xuan Thuy, Cau Giay, Hanoi, Vietnam; b Department of Chemistry, KU Leuven, Biomolecular Architecture, Celestijnenlaan 200F, Leuven (Heverlee), B-3001, Belgium; University of Aberdeen, United Kingdom

**Keywords:** crystal structure, platinum(II) complex, anti­cancer activity, catalytic activity

## Abstract

The title complex, [Pt(C_10_H_5_N_2_O_3_)Cl(C_18_H_33_P)], was synthesized by the reaction of [PtCl(C_10_H_5_N_2_O_3_)(η^2^-C_2_H_4_)] and PCy_3_ at room temperature for 2 h with a yield of 80%. The deprotonated 5-nitro­quinoline-8-ol (C_10_H_5_N_2_O_3_^−^) anion coordinates to the metal atom in a bidentate mode *via* its N and O atoms with a N—Pt—O bite angle of 80.0 (3)°. The tri­cyclo­hexyl­phosphine P atom is in a *trans* position with respect to the N atom in the square-planar coordination environment of the metal atom. The packing features zigzag chains linked by C—H⋯O inter­actions and continuous channels occupied by disordered solvent mol­ecules, both running in the *a*-axis direction. The complex shows weak activity against four cancer cell lines with IC_50_ values > 120 µ*M*, but significant catalytic ability and selectivity for hydro­silylation between phenyl­acetyl­ene and tri­ethyl­silane.

## Abstract 2

In the title square-planar complex, the P atom of the tri­cyclo­hexyl­phosphine group coordinates to the metal atom in the *trans* position compared to the coordinating N atom. The complex shows significant catalytic ability and selectivity for hydro­silylation between phenyl­acetyl­ene and tri­ethyl­silane.

## 1. Chemical context

In addition to several well-known platinum(II) complexes such as cisplatin, carboplatin, and oxaliplatin that are widely used in chemotherapy, numerous recent studies have highlighted the potential of Pt^II^-chelating complexes as promising catalysts in hydro­silylation, one of the most important reactions in the silicon industry (Stachowiak-Dłużyńska *et al.*, 2025 ▸; Walczak *et al.*, 2019 ▸; Thong *et al.*, 2024 ▸; Afanasenko *et al.*, 2020 ▸). In addition, several transition-metal complexes containing tri­cyclo­hexyl­phosphine (C_18_H_33_P, PCy_3_) are prominent catalysts in organic synthesis, such as the Grubbs and Crabtree catalysts (Trnka & Grubbs, 2000 ▸; Wüstenberg & Pfaltz, 2007 ▸). Recently, a number of Pt^II^ complexes bearing N,O-donor ligands (N^\O) and phosphine derivatives have been synthesized and evaluated for their anti­cancer activities (Živković *et al.*, 2018 ▸; Hyeraci *et al.*, 2020 ▸; Belli Dell’Amico *et al.*, 2018 ▸). However, their catalytic activity has not yet been explored.
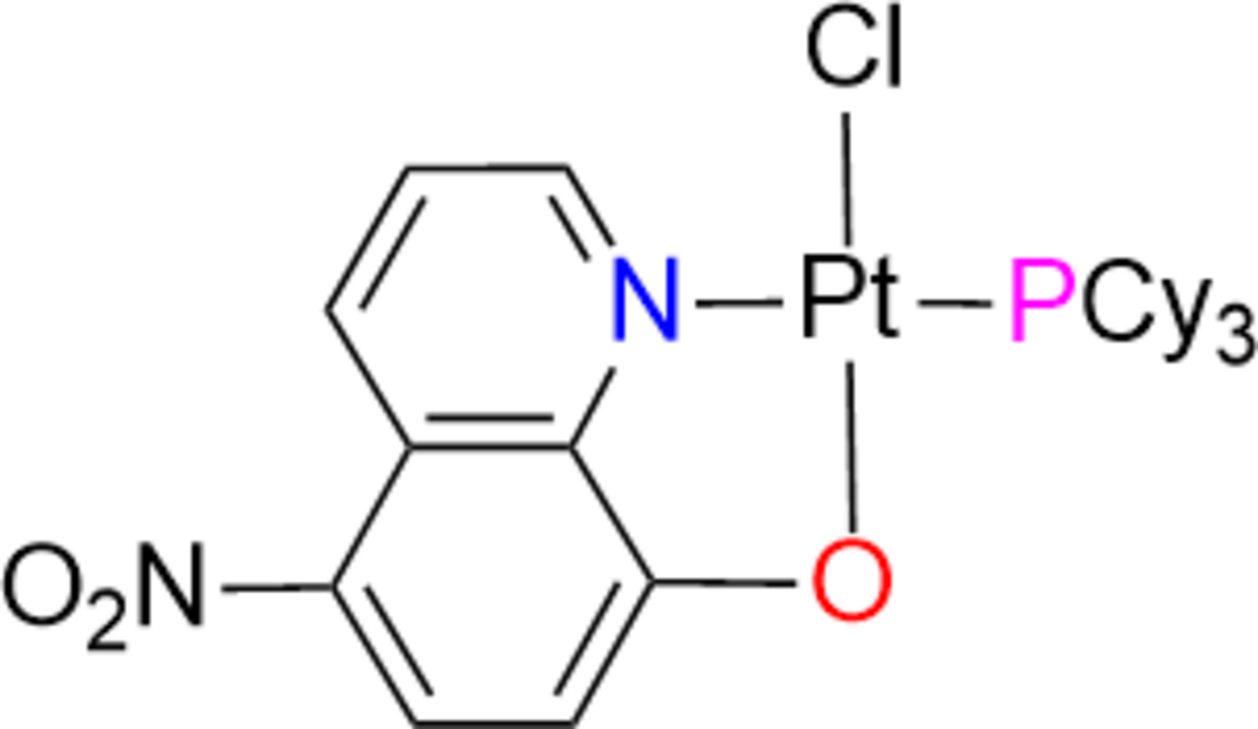


**Figure 1:**
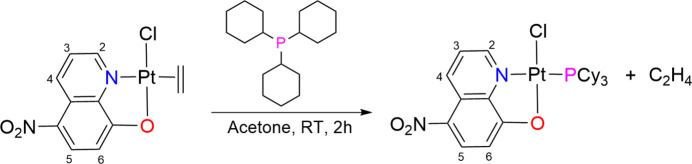
Synthesis scheme for the title complex.

In this study, a Pt^II^ complex containing the bidentate N^\O-type ligand 5-nitro­quinolin-8-ol (C_10_H_5_N_2_O_3_^−^ or NO_2_-HOQ), and PCy_3_ was synthesized. The reaction was carried out in acetone as the solvent, with the molar ratio of the complex [PtCl(NO_2_OQ)(η^2^-C_2_H_4_)]: PCy_3_ being 1:1 (Fig. 1 ▸) at room temperature for 2 h to form the title complex with a yield of 80%. The reactions proceeded rapidly under such mild conditions as the ethyl­ene ligand in the gas phase was quickly displaced by PR_3_ and evaporated from the reaction mixture.

**Figure 2:**
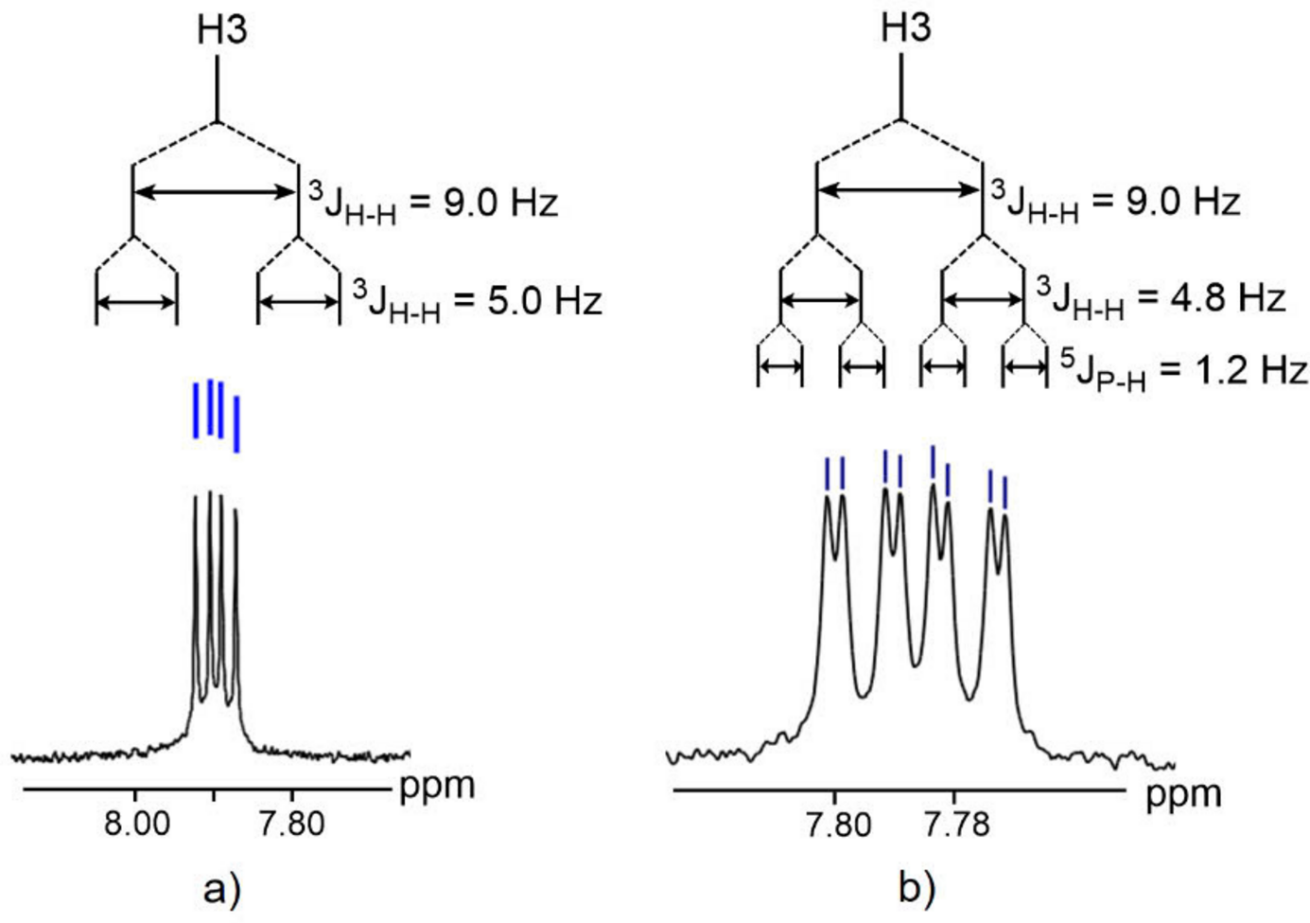
H3 signal in the ^1^H NMR spectra of (*a*) [PtCl(NO_2_OQ)(C_2_H_4_)] and (*b*) [PtCl(NO_2_OQ)(PCy_3_)].

The IR spectrum of the complex (Fig. S1) displays all characteristic vibrational bands for the functional groups present in the complex. For example, the characteristic signals for CH_aliphatic_ in PCy_3_ and CH_aromatic_ in NO_2_OQ appear in the region around 3000 cm^−1^. Meanwhile, two strong bands at 1504 and 1298 cm^−1^ correspond to ν_as_ and ν_s_ of the NO_2_ group in NO_2_OQ. The positive-mode ESI-MS spectrum (Fig. S2) exhibits a fragment with 100% relative intensity and an isotopic pattern consistent with the ion [Pt(NO_2_OQ)(PCy_3_)(CH_3_CN)]^+^ at *m*/*z* = 705. The formation of this fragment is attributed to the dissociation of the chloride ligand, followed by the coordination of a CH_3_CN mol­ecule to the Pt^II^ center. In the ^1^H NMR spectrum (Fig. S3), all the expected signals corresponding to the H atoms in PCy_3_ and NO_2_OQ are observed. Notably, several signals exhibit changes in chemical shift and/or shape compared to those of the free ligands and the starting complex. For example, the signals corresponding to H2, H3 and H6 in [PtCl(NO_2_OQ)(C_2_H_4_)] are *dd*, *dd* and *d*, respectively (Ly *et al.*, 2024 ▸), whereas in the complex they are *ddd*, *ddd* and *dd* (see Section 7 and Fig. 2 ▸). This change arises because these protons are coupled not only to the protons but also to ^31^P with ^4^*J*(P,H) = 4.8 Hz and ^5^*J*(P,H) = 1.2 Hz. These observations provide evidence for the coordination of PCy_3_ to Pt^II^*via* the P atom and of NO_2_OQ to Pt^II^ through both the N and O atoms.

## 2. Structural commentary

**Figure 3:**
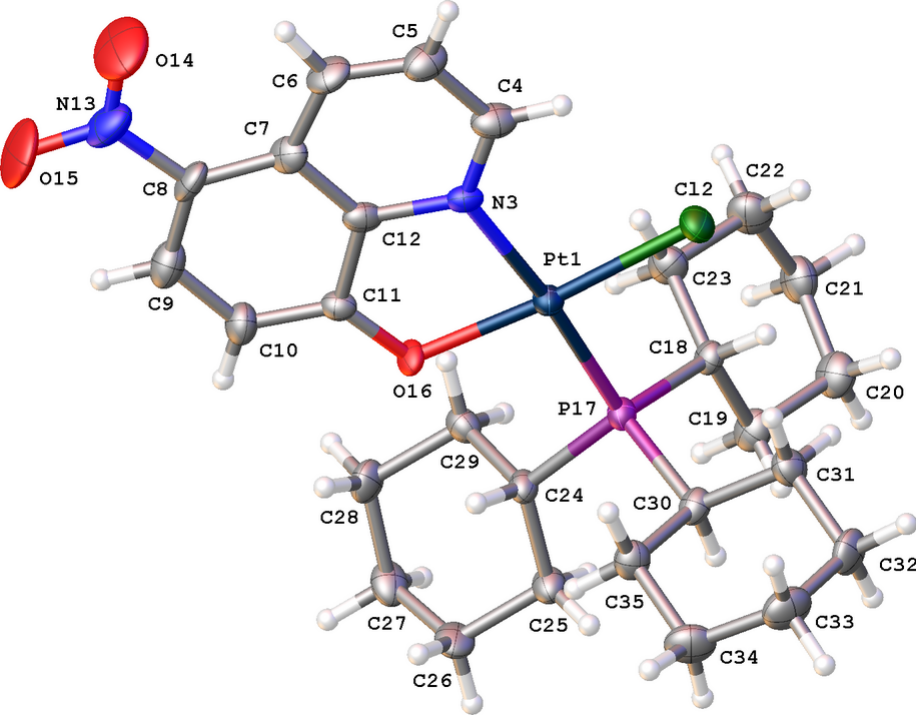
The mol­ecular structure of the title compound, showing displacement ellipsoids drawn at the 30% probability level.

The title complex crystallizes in the ortho­rhom­bic space group *P*2_1_2_1_2_1_ with one mol­ecule in the asymmetric unit (Fig. 3 ▸). The central Pt^II^ atom displays a square-planar coordination with one Cl atom, the N and O atoms of the quinolin-8-olate anion and the P atom of the PCy_3_ ligand and a τ(4) parameter of 0.08 (Yang *et al.*, 2007 ▸). The Pt^II^ atom deviates by 0.021 Å from the best plane through atoms N3, Cl2, O16 and P17 (r.m.s. deviation = 0.027 Å). The tri­cyclo­hexyl­phosphine (PCy_3_) ligand is in *trans* position with respect to the N atom. The three cyclo­hexyl groups have their usual chair conformation. The quinoline ring is almost planar with an r.m.s. deviation of 0.030 Å. Short intra­molecular C—H⋯Cl and C—H⋯O contacts are observed (Table 1 ▸).

## 3. Supra­molecular features

**Figure 4:**
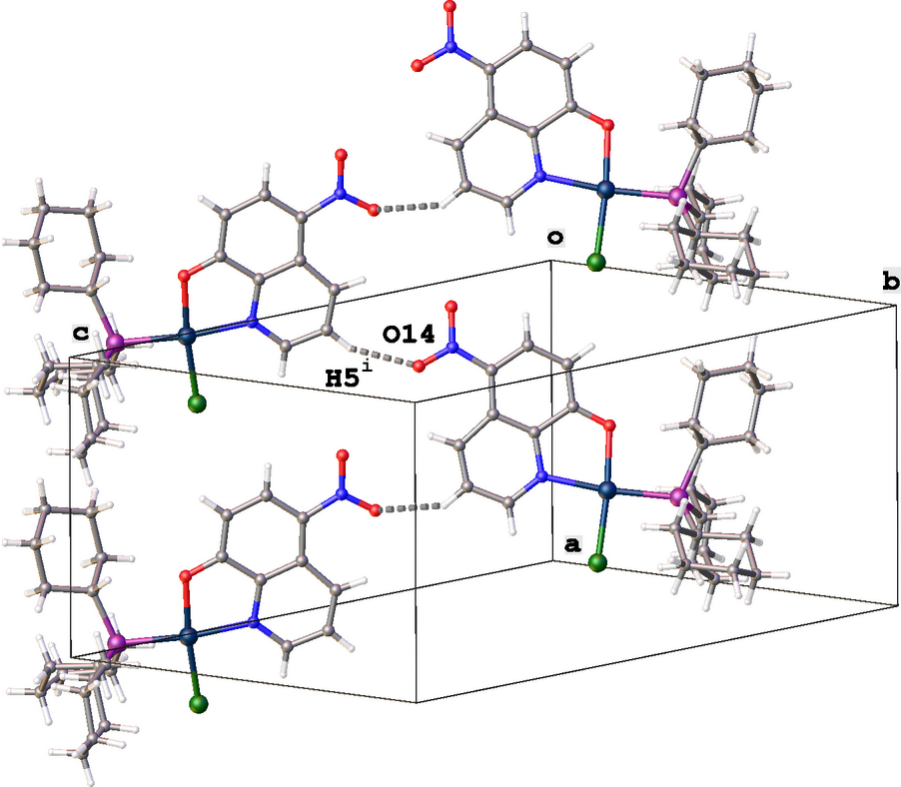
Partial packing diagram for the title compound showing the zigzag chain formed by C—H⋯O inter­actions [symmetry code: (i) *x* + 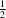, −*y* + 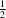, −*z* + 1].

**Figure 5:**
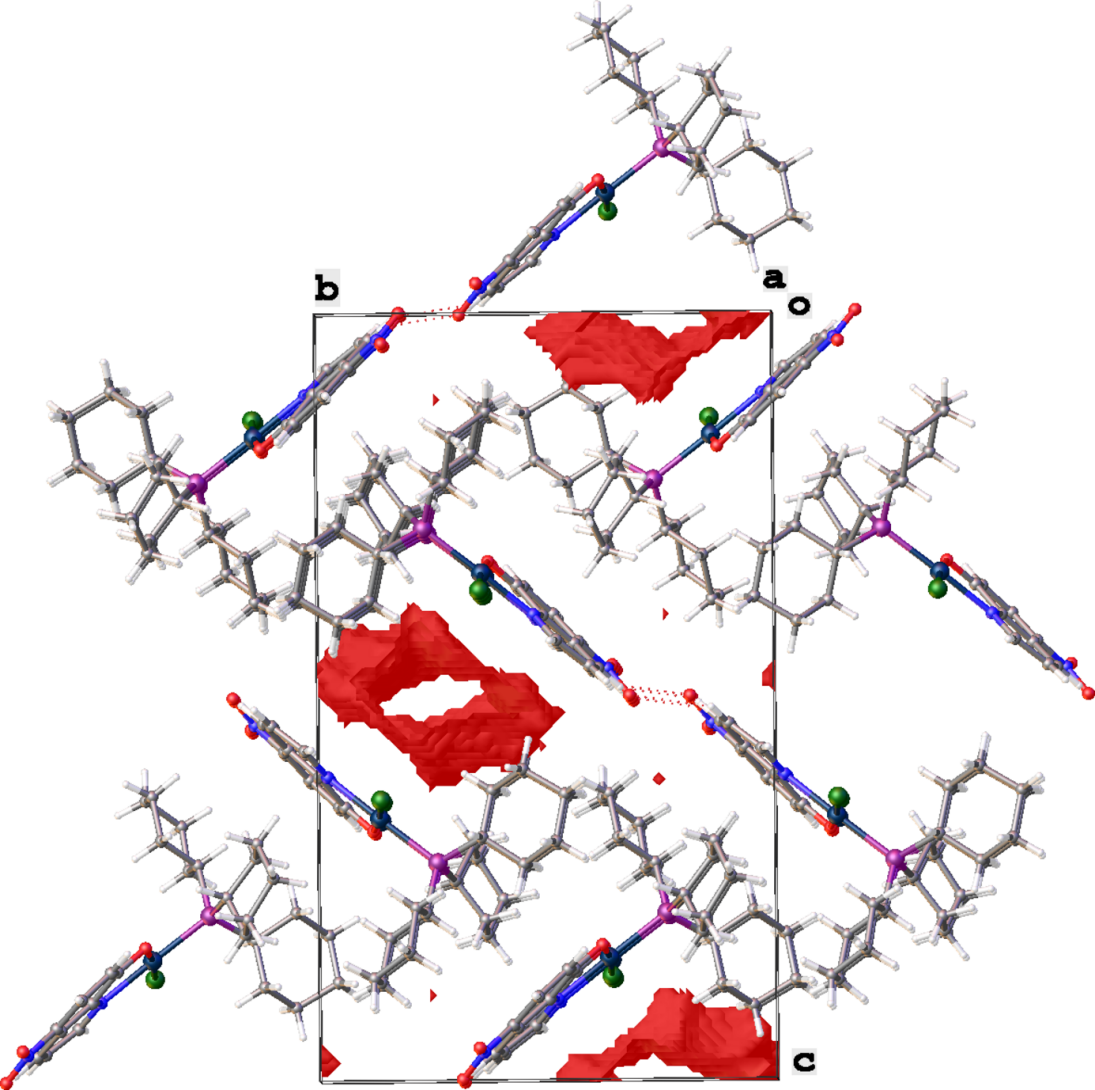
A view along the *a*-axis direction showing the continuous voids in the crystal packing of the title compound.

In the packing of the title complex, mol­ecules are linked by C5—H5⋯O14 inter­actions to form zigzag chains running in the *a*-axis direction (Table 1 ▸, Fig. 4 ▸). Between parallel chains are continuous channels with the quinoline and cyclo­hexyl groups acting as walls (Fig. 5 ▸). The packing index (percent filled space) is 58.2%. The disordered solvent (ethanol and/or acetone) in these chanels could not be located. Therefore, the solvent mask protocol in *OLEX2* (Dolomanov *et al.*, 2009 ▸) was used to account for the electron density present in the voids. No further significant inter­actions are observed in the crystal packing.

## 4. Database survey

A search of the Cambridge Structural Database (CSD, Version 6.00, update of April 2025; Groom *et al.*, 2016 ▸) for Pt complexes coordinated by Cl, N, O and P atoms resulted in 18 hits. In 11 structures, the P and N atoms are in a *trans* position with respect to each other. In all the structures, the metal atom displays a square-planar coordination with the Pt^II^ atom deviating between 0.002 and 0.066 Å from the best plane through the Cl, N, O and P atoms. The average Pt—Cl (2.299 Å), Pt—N (2.041 Å), Pt—O (2.033 Å) and Pt—P (2.224 Å) distances agree well with the corresponding distances in the title compound, which are 2.288 (2) Å, 2.087 (8) Å, 2.019 (6) Å and 2.249 (2) Å, respectively.

For the following structures, the N and O atoms are part of 8-hy­droxy­quinoline: chloro­(5-chloro-7-iodo­quinolin-8-olato)(1,3,5-tri­aza-7-phosphatri­cyclo­[3.3.1.1^3,7^]deca­ne)platinum (CSD refcode ZENVOG; Živković *et al.*, 2018 ▸) and chloro­(5,7-di­iodo­quinolin-8-olato)(1,3,5-tri­aza-7-phosphatri­cyclo­[3.3.1.1^3,7^]deca­ne)platinum (ZENVUM; Živković *et al.*, 2018 ▸). In none of the structures is the P atom part of tri­cyclo­hexyl­phosphine.

## 5. Catalytic tests

To evaluate the catalytic ability of the title compound for the hydro­silylation of phenyl­acetyl­ene by tri­ethyl­silane, a mixture of the complex (0.5 mol%), tri­ethyl­silane (1.0 mmol, 1.0 equiv) and phenyl­acetyl­ene (1.2 mmol, 1.2 equiv) was added to a Schlenk tube without solvent under air. The Schlenk tube was immersed in an oil bath preheated to the investigated temperatures (Table 2 ▸). After each predetermined reaction time (Table 2 ▸), the Schlenk tube was removed from the oil bath and the reaction mixture was allowed to cool to room temperature. The yields and molar ratios of the resulting products were determined by ^1^H NMR spectroscopy. The hydro­silylation reaction and the results are shown in Table 2 ▸.

**Figure 6:**
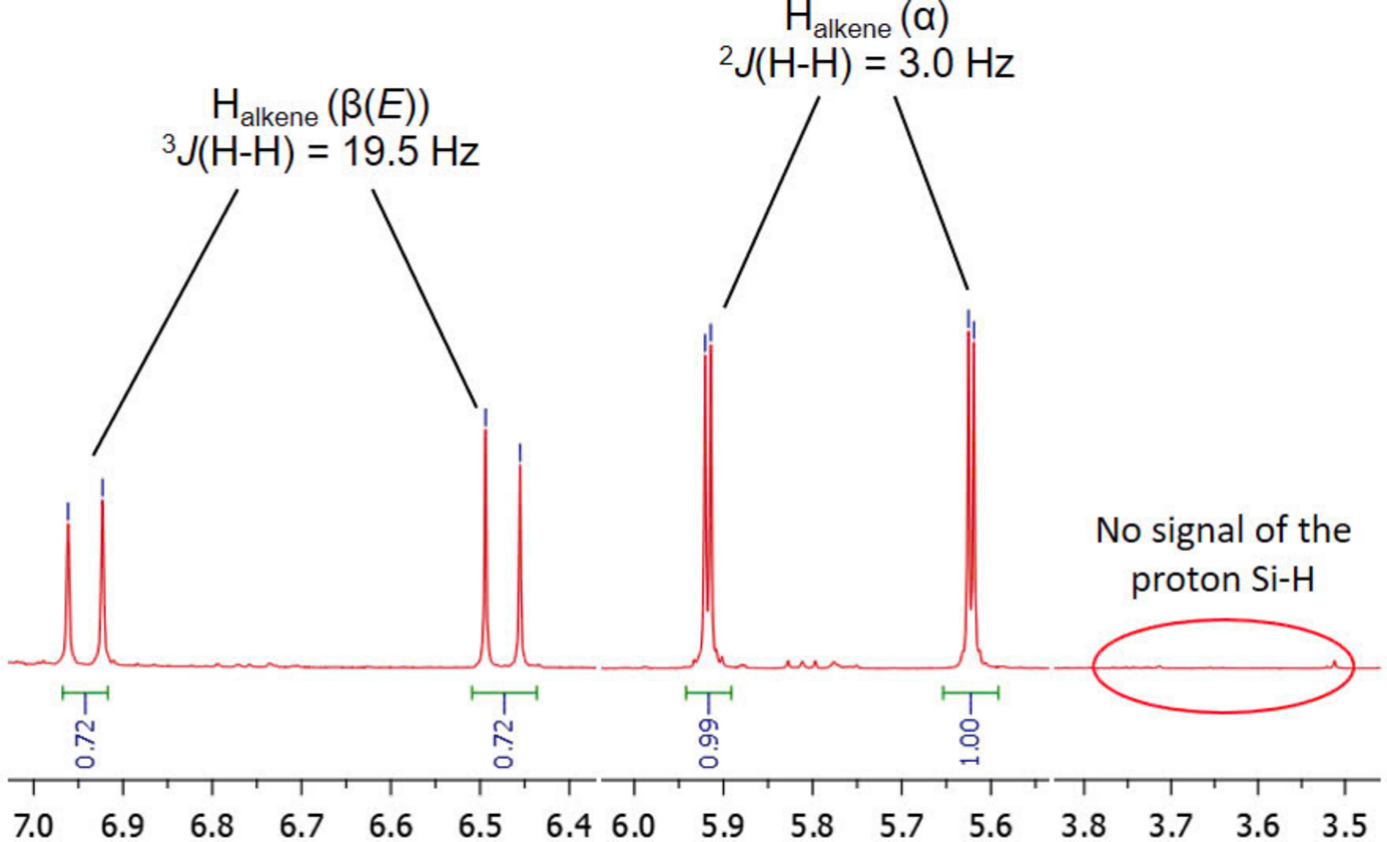
Partial ^1^H NMR spectrum of the product from entry 1 (Table 2 ▸) in chloro­form-*d*_1_.

The reaction conversion was verified by the signal intensity of the Si-H in tri­ethyl­silane. The regiochemistry and stereochemistry of the resulting alkenylsilane isomers were determined using olefinic coupling constants (Jun & Crabtree, 1993 ▸). For example, the absence of the Si-H signal at 3.62 ppm in the ^1^H NMR spectrum of the product from entry 1, run at 373 K for 5 h, confirms the complete consumption of tri­ethyl­silane (Fig. 6 ▸). The β(*E*)-isomer is identified by two doublets at 6.49 and 6.95 ppm with ^3^*J*_H–H_ = 19.5 Hz, corresponding to the CH=CH protons. In contrast, the α-isomer displays two geminal =CH_2_ protons as doublets at 5.62 and 5.92 ppm, with a ^2^*J*_H–H_ value of 3 Hz. From the integral of H_alkene_ signals of α and β(*E*), the α/β(*E*) molar ratio is determined for entry 1 to be 1.3:1.

The results in Table 2 ▸ indicate that the reaction temperature in entries 2 and 3 was reduced to 363 K while maintaining conversion of 100% and a constant α/β(*E*) molar ratio of 1:1.1 after 5 h (entry 2) and 3 h (entry 3). Therefore, in entry 4, both the reaction temperature was further decreased to 343 K and the reaction time was shortened to 2 h. The conversion significantly dropped to 57%. Notably, the product selectivity shifted markedly toward the β(*E*) isomer, with an α/β ratio of 1:2.3.

These results demonstrate that the title compound exhibits good catalytic activity for the hydro­silylation of phenyl­acetyl­ene by tri­ethyl­silane under mild conditions. Compared with several other Pt(II) complexes previously reported for this hydro­silylation reaction (Naganawa *et al.*, 2019 ▸; Fotie *et al.*, 2020 ▸; Afanasenko *et al.*, 2020 ▸), the title compound shows better catalytic activity. For instance, when di­chloro­(ethyl­enedi­amine)­platinum(II) was used as a catalyst at 1 mol% loading, only 75% conversion of tri­ethyl­silane was achieved after 18 h at 363 K (Fotie *et al.*, 2020 ▸).

## 6. *In vitro* cytotoxicity

The cytotoxicity of the Pt^II^ complex was evaluated against four human cancer cell lines, including epidermoid carcinoma (KB), lung cancer (Lu-1), hepatocellular carcinoma (Hep-G2), and breast cancer (MCF-7). Unfortunately, the results revealed that the title compound exhibits weak cytotoxic activity toward all tested cell lines, with IC_50_ values exceeding 120 µ*M*.

## 7. Synthesis and crystallization

A solution of tri­cyclo­hexyl­phosphine (28 mg, 0.1 mmol) in acetone was added dropwise to a solution of [PtCl(NO_2_OQ)(C_2_H_4_)] (44.75 mg, 0.1 mmol), prepared according to our previous reported procedure (Ly *et al.*, 2024 ▸), in the same solvent. The reaction mixture was stirred at room temperature: the evolution of gas bubbles was observed. After stirring for 30 minutes, a yellow–green precipitate began to form. This mixture was stirred for another 2 h. The resulting precipitate was collected by filtration and washed twice with 1 ml portions of cold ethanol. The title complex was obtained as a yellow–green solid in 80% yield. Crystals suitable for X-ray diffraction were obtained by slow evaporation from a saturated solution in the mixed solvents acetone/ethanol (*v*/*v* = 1:1) at room temperature.

^1^H NMR (600 MHz, chloro­form-*d_1_*): δ 9.68 [*dd*, ^3^*J*(H,H) = 9.0 Hz, ^5^*J*(P,H) = 1.2 Hz, H6], 9.21 [*ddd*, ^4^*J*(P,H) = 4.8 Hz, ^3^*J*(H,H) = 4.2 Hz, ^4^*J*(H,H) = 1.2 Hz, H2], 8.6 [*d*, ^3^*J*(H,H) = 9.6 Hz, H5], 7.79 [*ddd*, ^3^*J*(H,H) = 9.0 Hz, ^3^*J*(H,H) *=* 4.8 Hz, ^5^*J*(P,H) = 1.2 Hz, H3], 6.83 [*d*, ^3^*J*(H,H) = 9.0 Hz, H4], 2.42 (*m*, 3H, P-CH), 2.09–1.33 (30H, 10 CH_2_). + ESI MS (*m*/*z*, intensity): calculated for [*M* – Cl + CH_3_CN]^+^, C_29_H_41_N_3_O_3_PPt, 705, found 705, 100%. FT-IR (KBr pellet, cm^−1^): 2973, 2922 (CH), 1600, 1569, 1460 (C=C, C=N), 1504, 1298 (N=O).

## 8. Refinement

Crystal data, data collection and structure refinement details are summarized in Table 3 ▸. All hydrogen atoms were included as riding contributions in idealized positions with isotropic displacement parameters *U*_iso_(H) = 1.2 *U*_eq_(C). Anisotropic displacement parameters for the nitro atoms N13, O14 and O15 were refined with enhanced rigid bond (RIGU) restraints. The solvent mask protocol in *OLEX2* (Dolomanov *et al.*, 2009 ▸) was used to account for the void electron density corresponding to the disordered solvent mol­ecules (54 electrons in 144 Å^3^ void space per asymmetric unit). The structure was refined as an inversion twin [BASF = 0.349 (12)].

## Supplementary Material

Crystal structure: contains datablock(s) I. DOI: 10.1107/S2056989025005766/hb8142sup1.cif

Structure factors: contains datablock(s) I. DOI: 10.1107/S2056989025005766/hb8142Isup2.hkl

Supplementary spectroscopic data (IR, ESI MS, H-NMR). DOI: 10.1107/S2056989025005766/hb8142sup3.pdf

CCDC reference: 2467161

Additional supporting information:  crystallographic information; 3D view; checkCIF report

## Figures and Tables

**Table 1:** Hydrogen-bond geometry (Å, °)

*D*—H⋯*A*	*D*—H	H⋯*A*	*D*⋯*A*	*D*—H⋯*A*
C4—H4⋯Cl2	0.93	2.82	3.362 (12)	118
C5—H5⋯O14^i^	0.93	2.56	3.290 (17)	136
C6—H6⋯O14	0.93	2.25	2.809 (17)	118
C9—H9⋯O15	0.93	2.35	2.695 (17)	101
C24—H24⋯O16	0.98	2.45	2.985 (12)	114
C29—H29⋯O16	0.97	2.56	3.124 (12)	117
C31—H31⋯Cl2	0.97	2.83	3.500 (12)	127

Symmetry code: (i) 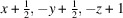.

**Table 2:** Performance of the Pt^II^ complex in the hydro­silylation reaction

Entry	Time (*h*)	Temperature (K)	Conversion (%)	α/β(*E*) ratio
1	5	373	100	1.3:1
2	5	363	100	1:1.1
3	3	363	100	1:1.1
4	2	343	57	1:2.3

**Table 3:** Experimental details

Crystal data	
Chemical formula	[Pt(C_10_H_5_N_2_O_3_)Cl(C_18_H_33_P)]
*M*_r_	700.10
Crystal system, space group	Orthorhombic, *P*2_1_2_1_2_1_
Temperature (K)	294
*a*, *b*, *c* (Å)	9.5189 (3), 14.0309 (5), 23.5582 (10)
*V* (Å^3^)	3146.4 (2)
*Z*	4
Radiation type	Mo *K*α
μ (mm^−1^)	4.62
Crystal size (mm)	0.4 × 0.25 × 0.1

Data collection	
Diffractometer	SuperNova, Single source at offset/far, Eos
Absorption correction	Multi-scan (*CrysAlis PRO*; Rigaku OD, 2024 ▸)
*T*_min_, *T*_max_	0.604, 1.000
No. of measured, independent and observed [*I* > 2σ(*I*)] reflections	18127, 6400, 5703
*R*_int_	0.044
(sin θ/λ)_max_ (Å^−1^)	0.625

Refinement	
*R*[*F*^2^ > 2σ(*F*^2^)], *wR*(*F*^2^), *S*	0.040, 0.090, 1.02
No. of reflections	6400
No. of parameters	317
No. of restraints	9
H-atom treatment	H-atom parameters constrained
Δρ_max_, Δρ_min_ (e Å^−3^)	1.80, −0.56
Absolute structure	Refined as an inversion twin
Absolute structure parameter	0.349 (12)

Computer programs: *CrysAlis PRO* (Rigaku OD, 2024 ▸), *SHELXT2014/5* (Sheldrick, 2015*a* ▸), *SHELXL2016/4* (Sheldrick, 2015*b* ▸) and *OLEX2* (Dolomanov *et al.*, 2009 ▸).

## supplementary crystallographic information

### Chlorido(5-nitroquinolin-8-olato-κ2N,O)(tricyclohexylphosphine-κP)platinum(II) Crystal data

**Table:**

[Pt(C_10_H_5_N_2_O_3_)Cl(C_18_H_33_P)]	*D*_x_ = 1.478 Mg m^−^^3^
*M**_r_* = 700.10	Mo *K*α radiation, λ = 0.71073 Å
Orthorhombic, *P*2_1_2_1_2_1_	Cell parameters from 8247 reflections
*a* = 9.5189 (3) Å	θ = 3.7–26.5°
*b* = 14.0309 (5) Å	µ = 4.62 mm^−^^1^
*c* = 23.5582 (10) Å	*T* = 294 K
*V* = 3146.4 (2) Å^3^	Plate, yellow
*Z* = 4	0.4 × 0.25 × 0.1 mm
*F*(000) = 1392	

### Chlorido(5-nitroquinolin-8-olato-κ2N,O)(tricyclohexylphosphine-κP)platinum(II) Data collection

**Table:**

SuperNova, Single source at offset/far, Eos diffractometer	6400 independent reflections
Radiation source: micro-focus sealed X-ray tube, SuperNova (Mo) X-ray Source	5703 reflections with *I* > 2σ(*I*)
Mirror monochromator	*R*_int_ = 0.044
Detector resolution: 15.9566 pixels mm^-1^	θ_max_ = 26.4°, θ_min_ = 3.4°
ω scans	*h* = −11→11
Absorption correction: multi-scan (CrysAlisPro; Rigaku OD, 2024)	*k* = −17→15
*T*_min_ = 0.604, *T*_max_ = 1.000	*l* = −29→29
18127 measured reflections	

### Chlorido(5-nitroquinolin-8-olato-κ2N,O)(tricyclohexylphosphine-κP)platinum(II) Refinement

**Table:**

Refinement on *F*^2^	Hydrogen site location: inferred from neighbouring sites
Least-squares matrix: full	H-atom parameters constrained
*R*[*F*^2^ > 2σ(*F*^2^)] = 0.040	*w* = 1/[σ^2^(*F*_o_^2^) + (0.0444*P*)^2^] where *P* = (*F*_o_^2^ + 2*F*_c_^2^)/3
*wR*(*F*^2^) = 0.090	(Δ/σ)_max_ = 0.001
*S* = 1.02	Δρ_max_ = 1.80 e Å^−^^3^
6400 reflections	Δρ_min_ = −0.56 e Å^−^^3^
317 parameters	Absolute structure: Refined as an inversion twin
9 restraints	Absolute structure parameter: 0.349 (12)
Primary atom site location: dual	

### Chlorido(5-nitroquinolin-8-olato-κ2N,O)(tricyclohexylphosphine-κP)platinum(II) Special details

**Table:**

Geometry. All esds (except the esd in the dihedral angle between two l.s. planes) are estimated using the full covariance matrix. The cell esds are taken into account individually in the estimation of esds in distances, angles and torsion angles; correlations between esds in cell parameters are only used when they are defined by crystal symmetry. An approximate (isotropic) treatment of cell esds is used for estimating esds involving l.s. planes.
Refinement. Refined as a 2-component inversion twin

### Chlorido(5-nitroquinolin-8-olato-κ2N,O)(tricyclohexylphosphine-κP)platinum(II) Fractional atomic coordinates and isotropic or equivalent isotropic displacement parameters (Å^2^)

**Table:**

	*x*	*y*	*z*	*U*_iso_*/*U*_eq_	
Pt1	0.55978 (4)	0.63620 (3)	0.34114 (2)	0.03216 (12)	
Cl2	0.7930 (2)	0.6400 (2)	0.36450 (12)	0.0497 (6)	
N3	0.5132 (8)	0.5231 (6)	0.3955 (4)	0.0333 (19)	
C4	0.5971 (13)	0.4730 (8)	0.4294 (5)	0.052 (3)	
H4	0.692649	0.486709	0.430632	0.063*	
C5	0.5434 (13)	0.4008 (8)	0.4628 (5)	0.054 (3)	
H5	0.605313	0.363444	0.483764	0.065*	
C6	0.4079 (13)	0.3827 (7)	0.4662 (5)	0.054 (3)	
H6	0.376640	0.335016	0.490551	0.065*	
C7	0.3073 (12)	0.4349 (7)	0.4331 (5)	0.045 (3)	
C8	0.1628 (12)	0.4265 (8)	0.4302 (5)	0.048 (3)	
C9	0.0843 (12)	0.4816 (7)	0.3937 (5)	0.055 (3)	
H9	−0.012873	0.475057	0.393009	0.066*	
C10	0.1482 (11)	0.5462 (8)	0.3581 (5)	0.049 (3)	
H10	0.092154	0.582165	0.333936	0.059*	
C11	0.2877 (10)	0.5595 (6)	0.3568 (4)	0.034 (2)	
C12	0.3692 (11)	0.5034 (7)	0.3965 (5)	0.038 (2)	
N13	0.0849 (13)	0.3630 (9)	0.4652 (5)	0.075 (3)	
O14	0.1436 (13)	0.3150 (9)	0.5006 (6)	0.144 (6)	
O15	−0.0468 (12)	0.3531 (9)	0.4602 (6)	0.127 (5)	
O16	0.3523 (6)	0.6214 (5)	0.3255 (3)	0.0379 (18)	
P17	0.5832 (2)	0.76251 (17)	0.28312 (11)	0.0323 (6)	
C18	0.6829 (9)	0.8599 (7)	0.3156 (4)	0.035 (2)	
H18	0.780070	0.837097	0.318091	0.042*	
C19	0.6889 (12)	0.9513 (7)	0.2804 (5)	0.050 (3)	
H19A	0.596136	0.979626	0.277971	0.060*	
H19B	0.720679	0.936955	0.242181	0.060*	
C20	0.7929 (13)	1.0220 (8)	0.3096 (6)	0.059 (3)	
H20A	0.887038	0.995616	0.307849	0.070*	
H20B	0.793134	1.081538	0.288682	0.070*	
C21	0.7557 (12)	1.0421 (9)	0.3713 (6)	0.059 (3)	
H21A	0.828571	1.080833	0.388478	0.071*	
H21B	0.668062	1.077187	0.373102	0.071*	
C22	0.7413 (13)	0.9492 (9)	0.4037 (6)	0.063 (4)	
H22A	0.832468	0.918571	0.405946	0.076*	
H22B	0.710805	0.962961	0.442165	0.076*	
C23	0.6384 (11)	0.8816 (8)	0.3767 (5)	0.051 (3)	
H23A	0.545325	0.909748	0.376807	0.061*	
H23B	0.634658	0.822851	0.398394	0.061*	
C24	0.4064 (9)	0.7998 (7)	0.2600 (4)	0.037 (2)	
H24	0.358740	0.740569	0.249281	0.045*	
C25	0.3899 (10)	0.8650 (8)	0.2090 (5)	0.045 (3)	
H25A	0.421555	0.928664	0.218686	0.054*	
H25B	0.447373	0.841759	0.177934	0.054*	
C26	0.2382 (10)	0.8681 (9)	0.1909 (5)	0.052 (3)	
H26A	0.229343	0.909326	0.158057	0.063*	
H26B	0.208771	0.804644	0.179781	0.063*	
C27	0.1433 (11)	0.9035 (8)	0.2367 (5)	0.055 (3)	
H27A	0.046524	0.899051	0.224117	0.066*	
H27B	0.163777	0.970006	0.244341	0.066*	
C28	0.1616 (10)	0.8464 (8)	0.2904 (5)	0.050 (3)	
H28A	0.125738	0.782484	0.284571	0.060*	
H28B	0.107435	0.875641	0.320682	0.060*	
C29	0.3181 (10)	0.8410 (7)	0.3084 (5)	0.041 (3)	
H29A	0.351832	0.904220	0.317924	0.049*	
H29B	0.327242	0.801002	0.341781	0.049*	
C30	0.6759 (10)	0.7363 (7)	0.2172 (4)	0.038 (2)	
H30	0.668567	0.793530	0.193523	0.046*	
C31	0.8315 (10)	0.7153 (8)	0.2237 (5)	0.046 (3)	
H31A	0.843573	0.658591	0.246682	0.056*	
H31B	0.876596	0.768007	0.243094	0.056*	
C32	0.9022 (10)	0.7001 (8)	0.1656 (5)	0.054 (3)	
H32A	0.899445	0.759192	0.144230	0.065*	
H32B	0.999955	0.683078	0.171287	0.065*	
C33	0.8304 (13)	0.6229 (9)	0.1321 (5)	0.066 (4)	
H33A	0.871075	0.620620	0.094337	0.079*	
H33B	0.848241	0.562024	0.150196	0.079*	
C34	0.6754 (14)	0.6369 (10)	0.1269 (5)	0.064 (3)	
H34A	0.633846	0.580242	0.110338	0.076*	
H34B	0.657120	0.689882	0.101523	0.076*	
C35	0.6048 (11)	0.6569 (7)	0.1849 (5)	0.046 (3)	
H35A	0.507116	0.673720	0.178690	0.055*	
H35B	0.606947	0.599334	0.207654	0.055*	

### Chlorido(5-nitroquinolin-8-olato-κ2N,O)(tricyclohexylphosphine-κP)platinum(II) Atomic displacement parameters (Å^2^)

**Table:**

	*U*^11^	*U*^22^	*U*^33^	*U*^12^	*U*^13^	*U*^23^
Pt1	0.03246 (17)	0.02928 (18)	0.03475 (19)	0.00223 (18)	−0.00158 (19)	0.00474 (18)
Cl2	0.0359 (11)	0.0594 (16)	0.0536 (16)	0.0023 (14)	−0.0075 (11)	0.0146 (16)
N3	0.046 (5)	0.030 (4)	0.024 (5)	0.004 (4)	−0.002 (4)	0.000 (4)
C4	0.065 (8)	0.046 (6)	0.046 (7)	0.016 (6)	−0.001 (6)	0.007 (6)
C5	0.063 (7)	0.047 (6)	0.052 (7)	0.013 (6)	0.008 (7)	0.013 (6)
C6	0.075 (8)	0.036 (6)	0.050 (7)	−0.006 (6)	0.005 (6)	0.019 (5)
C7	0.058 (7)	0.029 (5)	0.047 (7)	−0.013 (5)	−0.003 (6)	0.000 (5)
C8	0.056 (7)	0.043 (6)	0.045 (7)	−0.017 (6)	0.021 (6)	0.003 (5)
C9	0.053 (7)	0.043 (6)	0.070 (9)	−0.008 (6)	0.018 (7)	−0.002 (6)
C10	0.041 (6)	0.047 (6)	0.060 (8)	−0.009 (5)	0.005 (5)	0.003 (6)
C11	0.042 (5)	0.028 (5)	0.032 (6)	0.002 (4)	−0.004 (4)	0.001 (4)
C12	0.049 (6)	0.038 (6)	0.029 (6)	0.010 (5)	0.000 (5)	−0.002 (5)
N13	0.101 (7)	0.068 (7)	0.057 (7)	−0.033 (8)	0.015 (6)	0.012 (6)
O14	0.130 (10)	0.137 (11)	0.164 (13)	−0.046 (8)	−0.006 (9)	0.104 (10)
O15	0.098 (7)	0.130 (10)	0.155 (12)	−0.070 (8)	0.032 (7)	0.044 (9)
O16	0.032 (3)	0.031 (4)	0.051 (5)	−0.011 (3)	−0.002 (3)	0.019 (3)
P17	0.0285 (12)	0.0292 (12)	0.0390 (15)	−0.0001 (10)	−0.0001 (11)	0.0045 (11)
C18	0.036 (4)	0.029 (4)	0.039 (5)	−0.007 (5)	0.003 (4)	0.003 (5)
C19	0.059 (7)	0.033 (5)	0.058 (8)	−0.009 (6)	0.006 (6)	−0.002 (5)
C20	0.063 (7)	0.041 (6)	0.071 (9)	−0.009 (6)	0.004 (7)	0.003 (6)
C21	0.053 (7)	0.048 (7)	0.077 (10)	−0.013 (6)	−0.001 (7)	−0.003 (7)
C22	0.070 (8)	0.066 (8)	0.054 (9)	−0.004 (7)	−0.003 (7)	−0.017 (7)
C23	0.057 (6)	0.046 (7)	0.049 (7)	−0.006 (5)	−0.002 (5)	−0.008 (6)
C24	0.028 (5)	0.039 (6)	0.044 (6)	−0.001 (4)	−0.002 (4)	0.012 (5)
C25	0.039 (5)	0.043 (6)	0.054 (7)	−0.004 (5)	−0.003 (5)	0.018 (6)
C26	0.045 (5)	0.055 (7)	0.057 (7)	0.011 (6)	−0.010 (5)	0.024 (7)
C27	0.040 (6)	0.046 (6)	0.081 (9)	0.007 (5)	−0.006 (6)	0.018 (7)
C28	0.038 (5)	0.054 (7)	0.058 (7)	0.004 (5)	0.016 (5)	0.001 (6)
C29	0.040 (5)	0.037 (6)	0.047 (7)	−0.003 (5)	−0.009 (5)	0.001 (5)
C30	0.038 (5)	0.030 (5)	0.046 (7)	0.001 (4)	0.008 (5)	0.006 (5)
C31	0.038 (5)	0.051 (6)	0.050 (7)	0.004 (5)	−0.002 (5)	0.001 (6)
C32	0.036 (5)	0.061 (7)	0.066 (9)	0.008 (5)	0.021 (6)	0.002 (7)
C33	0.075 (8)	0.072 (9)	0.052 (8)	0.018 (8)	0.018 (7)	−0.003 (7)
C34	0.085 (9)	0.062 (7)	0.044 (7)	0.009 (8)	−0.001 (6)	−0.013 (7)
C35	0.044 (5)	0.044 (7)	0.049 (7)	0.002 (5)	0.006 (5)	−0.006 (5)

### Chlorido(5-nitroquinolin-8-olato-κ2N,O)(tricyclohexylphosphine-κP)platinum(II) Geometric parameters (Å, º)

**Table:**

Pt1—Cl2	2.288 (2)	C22—H22A	0.9700
Pt1—N3	2.087 (8)	C22—H22B	0.9700
Pt1—O16	2.019 (6)	C22—C23	1.505 (14)
Pt1—P17	2.249 (2)	C23—H23A	0.9700
N3—C4	1.331 (13)	C23—H23B	0.9700
N3—C12	1.398 (12)	C24—H24	0.9800
C4—H4	0.9300	C24—C25	1.519 (13)
C4—C5	1.381 (15)	C24—C29	1.529 (14)
C5—H5	0.9300	C25—H25A	0.9700
C5—C6	1.317 (16)	C25—H25B	0.9700
C6—H6	0.9300	C25—C26	1.507 (13)
C6—C7	1.436 (16)	C26—H26A	0.9700
C7—C8	1.383 (16)	C26—H26B	0.9700
C7—C12	1.419 (14)	C26—C27	1.492 (15)
C8—C9	1.377 (16)	C27—H27A	0.9700
C8—N13	1.422 (14)	C27—H27B	0.9700
C9—H9	0.9300	C27—C28	1.508 (15)
C9—C10	1.375 (14)	C28—H28A	0.9700
C10—H10	0.9300	C28—H28B	0.9700
C10—C11	1.342 (13)	C28—C29	1.551 (13)
C11—C12	1.447 (13)	C29—H29A	0.9700
C11—O16	1.295 (11)	C29—H29B	0.9700
N13—O14	1.208 (15)	C30—H30	0.9800
N13—O15	1.267 (15)	C30—C31	1.518 (13)
P17—C18	1.832 (10)	C30—C35	1.509 (14)
P17—C24	1.844 (9)	C31—H31A	0.9700
P17—C30	1.823 (10)	C31—H31B	0.9700
C18—H18	0.9800	C31—C32	1.540 (15)
C18—C19	1.529 (14)	C32—H32A	0.9700
C18—C23	1.531 (14)	C32—H32B	0.9700
C19—H19A	0.9700	C32—C33	1.504 (16)
C19—H19B	0.9700	C33—H33A	0.9700
C19—C20	1.562 (15)	C33—H33B	0.9700
C20—H20A	0.9700	C33—C34	1.494 (17)
C20—H20B	0.9700	C34—H34A	0.9700
C20—C21	1.523 (17)	C34—H34B	0.9700
C21—H21A	0.9700	C34—C35	1.548 (14)
C21—H21B	0.9700	C35—H35A	0.9700
C21—C22	1.517 (17)	C35—H35B	0.9700
			
N3—Pt1—Cl2	94.4 (2)	C18—C23—H23B	109.7
N3—Pt1—P17	173.3 (2)	C22—C23—C18	110.0 (9)
O16—Pt1—Cl2	174.3 (2)	C22—C23—H23A	109.7
O16—Pt1—N3	80.0 (3)	C22—C23—H23B	109.7
O16—Pt1—P17	93.83 (19)	H23A—C23—H23B	108.2
P17—Pt1—Cl2	91.83 (9)	P17—C24—H24	105.0
C4—N3—Pt1	130.0 (8)	C25—C24—P17	119.9 (7)
C4—N3—C12	118.3 (10)	C25—C24—H24	105.0
C12—N3—Pt1	111.6 (7)	C25—C24—C29	107.8 (8)
N3—C4—H4	119.8	C29—C24—P17	112.9 (7)
N3—C4—C5	120.5 (11)	C29—C24—H24	105.0
C5—C4—H4	119.8	C24—C25—H25A	109.7
C4—C5—H5	118.7	C24—C25—H25B	109.7
C6—C5—C4	122.6 (12)	H25A—C25—H25B	108.2
C6—C5—H5	118.7	C26—C25—C24	109.9 (9)
C5—C6—H6	119.3	C26—C25—H25A	109.7
C5—C6—C7	121.5 (10)	C26—C25—H25B	109.7
C7—C6—H6	119.3	C25—C26—H26A	109.1
C8—C7—C6	130.3 (10)	C25—C26—H26B	109.1
C8—C7—C12	116.2 (10)	H26A—C26—H26B	107.8
C12—C7—C6	113.5 (10)	C27—C26—C25	112.7 (10)
C7—C8—N13	122.9 (12)	C27—C26—H26A	109.1
C9—C8—C7	121.5 (10)	C27—C26—H26B	109.1
C9—C8—N13	115.6 (11)	C26—C27—H27A	109.4
C8—C9—H9	119.6	C26—C27—H27B	109.4
C10—C9—C8	120.7 (11)	C26—C27—C28	111.1 (9)
C10—C9—H9	119.6	H27A—C27—H27B	108.0
C9—C10—H10	118.5	C28—C27—H27A	109.4
C11—C10—C9	122.9 (11)	C28—C27—H27B	109.4
C11—C10—H10	118.5	C27—C28—H28A	109.3
C10—C11—C12	116.1 (10)	C27—C28—H28B	109.3
O16—C11—C10	125.2 (9)	C27—C28—C29	111.5 (9)
O16—C11—C12	118.6 (8)	H28A—C28—H28B	108.0
N3—C12—C7	123.4 (10)	C29—C28—H28A	109.3
N3—C12—C11	114.0 (9)	C29—C28—H28B	109.3
C7—C12—C11	122.6 (9)	C24—C29—C28	110.1 (8)
O14—N13—C8	120.6 (13)	C24—C29—H29A	109.6
O14—N13—O15	117.4 (12)	C24—C29—H29B	109.6
O15—N13—C8	122.0 (13)	C28—C29—H29A	109.6
C11—O16—Pt1	115.4 (6)	C28—C29—H29B	109.6
C18—P17—Pt1	112.6 (3)	H29A—C29—H29B	108.2
C18—P17—C24	112.6 (5)	P17—C30—H30	106.6
C24—P17—Pt1	108.2 (3)	C31—C30—P17	115.2 (8)
C30—P17—Pt1	114.0 (3)	C31—C30—H30	106.6
C30—P17—C18	104.8 (4)	C35—C30—P17	111.2 (7)
C30—P17—C24	104.3 (5)	C35—C30—H30	106.6
P17—C18—H18	105.7	C35—C30—C31	110.2 (8)
C19—C18—P17	114.7 (7)	C30—C31—H31A	109.3
C19—C18—H18	105.7	C30—C31—H31B	109.3
C19—C18—C23	110.8 (9)	C30—C31—C32	111.4 (9)
C23—C18—P17	113.4 (7)	H31A—C31—H31B	108.0
C23—C18—H18	105.7	C32—C31—H31A	109.3
C18—C19—H19A	110.0	C32—C31—H31B	109.3
C18—C19—H19B	110.0	C31—C32—H32A	109.3
C18—C19—C20	108.5 (9)	C31—C32—H32B	109.3
H19A—C19—H19B	108.4	H32A—C32—H32B	108.0
C20—C19—H19A	110.0	C33—C32—C31	111.6 (9)
C20—C19—H19B	110.0	C33—C32—H32A	109.3
C19—C20—H20A	109.0	C33—C32—H32B	109.3
C19—C20—H20B	109.0	C32—C33—H33A	108.9
H20A—C20—H20B	107.8	C32—C33—H33B	108.9
C21—C20—C19	113.0 (10)	H33A—C33—H33B	107.7
C21—C20—H20A	109.0	C34—C33—C32	113.4 (10)
C21—C20—H20B	109.0	C34—C33—H33A	108.9
C20—C21—H21A	109.6	C34—C33—H33B	108.9
C20—C21—H21B	109.6	C33—C34—H34A	109.1
H21A—C21—H21B	108.2	C33—C34—H34B	109.1
C22—C21—C20	110.1 (11)	C33—C34—C35	112.4 (10)
C22—C21—H21A	109.6	H34A—C34—H34B	107.9
C22—C21—H21B	109.6	C35—C34—H34A	109.1
C21—C22—H22A	109.0	C35—C34—H34B	109.1
C21—C22—H22B	109.0	C30—C35—C34	112.6 (9)
H22A—C22—H22B	107.8	C30—C35—H35A	109.1
C23—C22—C21	112.8 (11)	C30—C35—H35B	109.1
C23—C22—H22A	109.0	C34—C35—H35A	109.1
C23—C22—H22B	109.0	C34—C35—H35B	109.1
C18—C23—H23A	109.7	H35A—C35—H35B	107.8
			
Pt1—N3—C4—C5	179.2 (8)	O16—C11—C12—C7	179.1 (9)
Pt1—N3—C12—C7	−175.0 (8)	P17—C18—C19—C20	−172.6 (7)
Pt1—N3—C12—C11	4.8 (11)	P17—C18—C23—C22	169.8 (8)
Pt1—P17—C18—C19	−174.0 (7)	P17—C24—C25—C26	167.8 (8)
Pt1—P17—C18—C23	−45.4 (8)	P17—C24—C29—C28	−165.5 (7)
Pt1—P17—C24—C25	−163.5 (8)	P17—C30—C31—C32	−176.6 (7)
Pt1—P17—C24—C29	67.8 (7)	P17—C30—C35—C34	176.5 (8)
Pt1—P17—C30—C31	−69.4 (8)	C18—P17—C24—C25	71.3 (10)
Pt1—P17—C30—C35	56.8 (8)	C18—P17—C24—C29	−57.4 (8)
N3—C4—C5—C6	−5.2 (19)	C18—P17—C30—C31	54.2 (8)
C4—N3—C12—C7	2.1 (16)	C18—P17—C30—C35	−179.6 (7)
C4—N3—C12—C11	−178.2 (9)	C18—C19—C20—C21	−55.4 (13)
C4—C5—C6—C7	2.7 (19)	C19—C18—C23—C22	−59.6 (11)
C5—C6—C7—C8	179.4 (12)	C19—C20—C21—C22	53.4 (13)
C5—C6—C7—C12	1.9 (17)	C20—C21—C22—C23	−54.5 (13)
C6—C7—C8—C9	−177.4 (11)	C21—C22—C23—C18	57.9 (13)
C6—C7—C8—N13	4 (2)	C23—C18—C19—C20	57.5 (11)
C6—C7—C12—N3	−4.3 (16)	C24—P17—C18—C19	−51.2 (9)
C6—C7—C12—C11	176.0 (9)	C24—P17—C18—C23	77.4 (8)
C7—C8—C9—C10	0.9 (18)	C24—P17—C30—C31	172.8 (7)
C7—C8—N13—O14	2 (2)	C24—P17—C30—C35	−61.0 (8)
C7—C8—N13—O15	−176.7 (13)	C24—C25—C26—C27	59.5 (13)
C8—C7—C12—N3	177.8 (10)	C25—C24—C29—C28	59.8 (11)
C8—C7—C12—C11	−1.9 (16)	C25—C26—C27—C28	−54.3 (14)
C8—C9—C10—C11	−0.1 (18)	C26—C27—C28—C29	52.1 (13)
C9—C8—N13—O14	−176.5 (14)	C27—C28—C29—C24	−56.1 (12)
C9—C8—N13—O15	4.9 (19)	C29—C24—C25—C26	−61.2 (12)
C9—C10—C11—C12	−1.6 (16)	C30—P17—C18—C19	61.6 (8)
C9—C10—C11—O16	−177.7 (10)	C30—P17—C18—C23	−169.8 (7)
C10—C11—C12—N3	−177.1 (10)	C30—P17—C24—C25	−41.8 (10)
C10—C11—C12—C7	2.7 (15)	C30—P17—C24—C29	−170.5 (7)
C10—C11—O16—Pt1	172.0 (9)	C30—C31—C32—C33	−55.6 (12)
C12—N3—C4—C5	2.7 (16)	C31—C30—C35—C34	−54.5 (12)
C12—C7—C8—C9	0.1 (17)	C31—C32—C33—C34	52.1 (14)
C12—C7—C8—N13	−178.3 (10)	C32—C33—C34—C35	−49.8 (15)
C12—C11—O16—Pt1	−4.1 (11)	C33—C34—C35—C30	51.3 (15)
N13—C8—C9—C10	179.4 (11)	C35—C30—C31—C32	56.7 (12)
O16—C11—C12—N3	−0.7 (14)		

### Chlorido(5-nitroquinolin-8-olato-κ2N,O)(tricyclohexylphosphine-κP)platinum(II) Hydrogen-bond geometry (Å, º)

**Table:**

*D*—H···*A*	*D*—H	H···*A*	*D*···*A*	*D*—H···*A*
C4—H4···Cl2	0.93	2.82	3.362 (12)	118
C5—H5···O14^i^	0.93	2.56	3.290 (17)	136
C6—H6···O14	0.93	2.25	2.809 (17)	118
C9—H9···O15	0.93	2.35	2.695 (17)	101
C24—H24···O16	0.98	2.45	2.985 (12)	114
C29—H29···O16	0.97	2.56	3.124 (12)	117
C31—H31···Cl2	0.97	2.83	3.500 (12)	127

Symmetry code: (i) *x*+1/2, −*y*+1/2, −*z*+1.
