# Supplementary material for: Synthesis, structure, catalytic and cytotoxic activities of chlorido­(5-nitro­quinolin-8-olato-κ2N,O)(tri­cyclo­hexyl­phosphine-κP)platinum(II)
Source: Acta Crystallogr E Crystallogr Commun. 2025 Jul 1;81(Pt 8):657–61. doi: 10.1107/S2056989025005766 (PMC12326502; doi:10.1107/S2056989025005766)
Supplement: Supplementary file 3 [file e-81-00657-sup3.pdf]

Supplementary Information

Synthesis, crystal structure, catalytic and cytotoxic activities of complex [PtCl(NO$_2$OQ)(PCy$_3$)]

Nguyen Thi Thanh Chi,[a] Nguyen Tran Huong Ly,[a] Duc Doan Hieu,[a] Luc Van Meervelt [b]

[a] *Faculty of Chemistry, Hanoi National University of Education, Hanoi, 10000 Vietnam.*

[b]*Department of Chemistry, KU Leuven, Celestijnenlaan 200F, B-3001 Leuven, Belgium.*

Correspondence email: luc.vanmeervelt@kuleuven.be, chintt@hnue.edu.vn

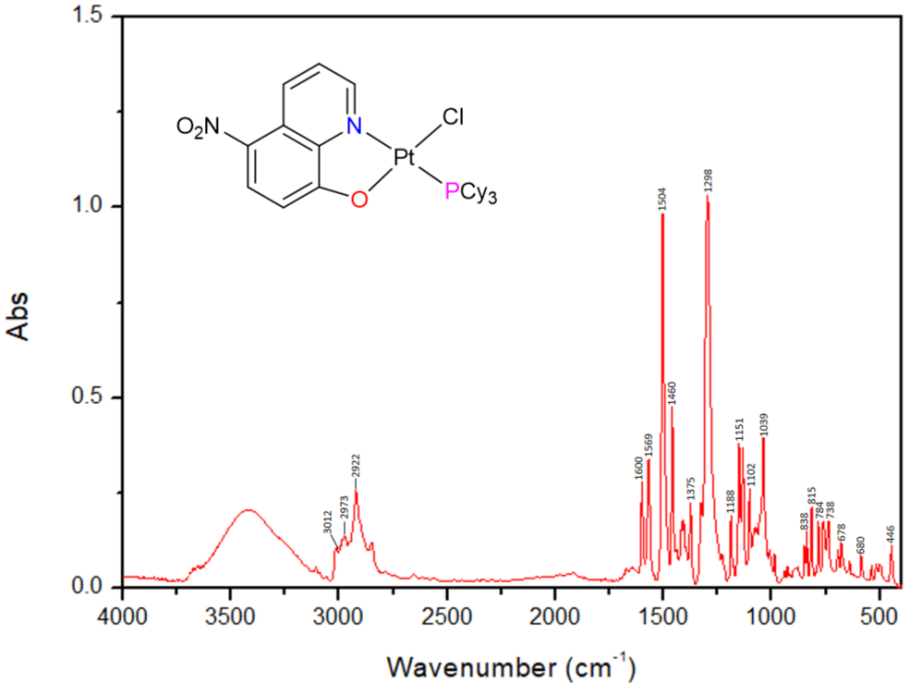

Figure S1. IR spectrum of (I)

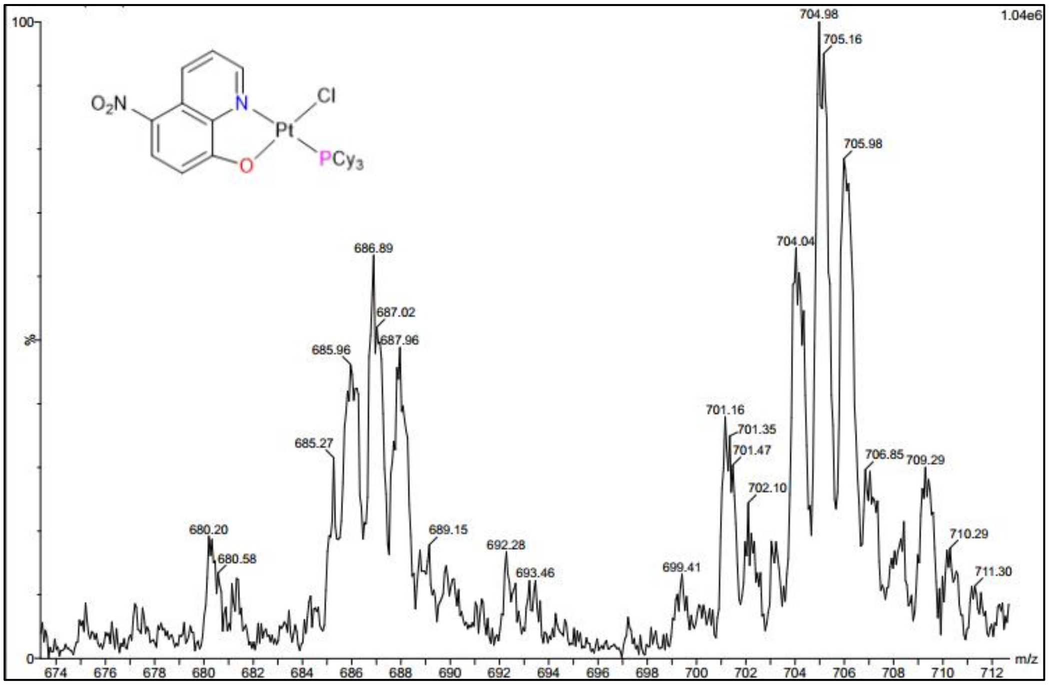

Figure S2. +ESI mass spectrum of (I)

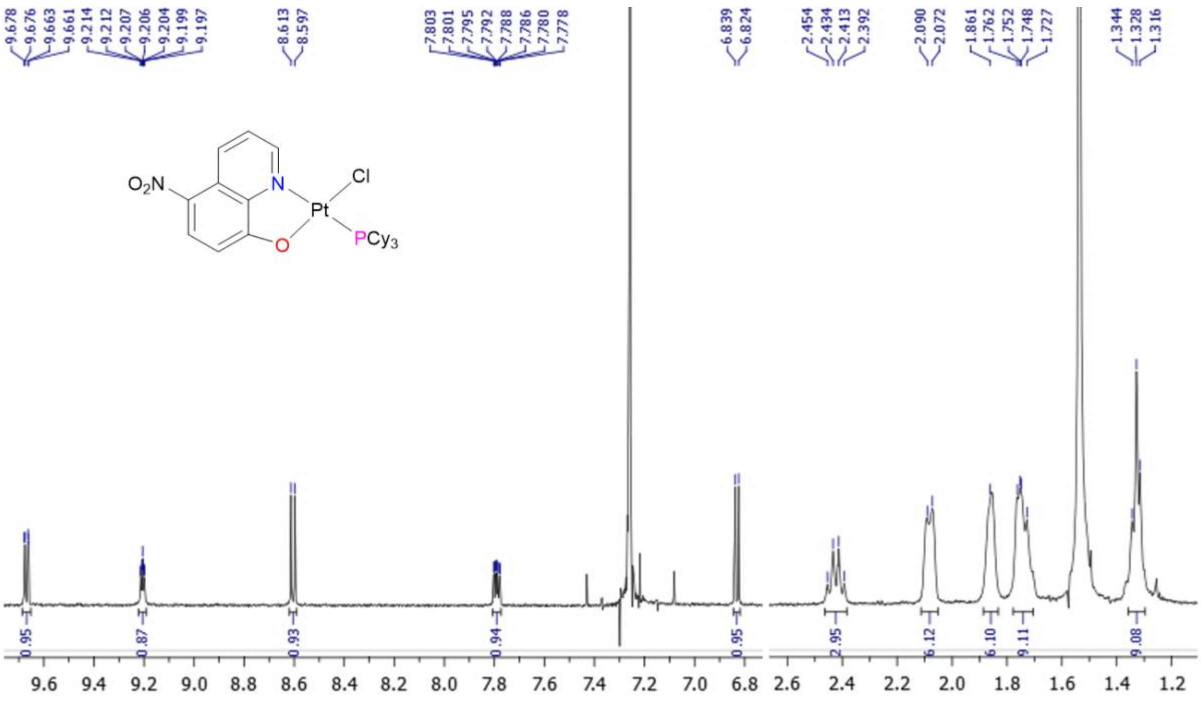

Figure S3. ^{1}H NMR spectrum of (I)

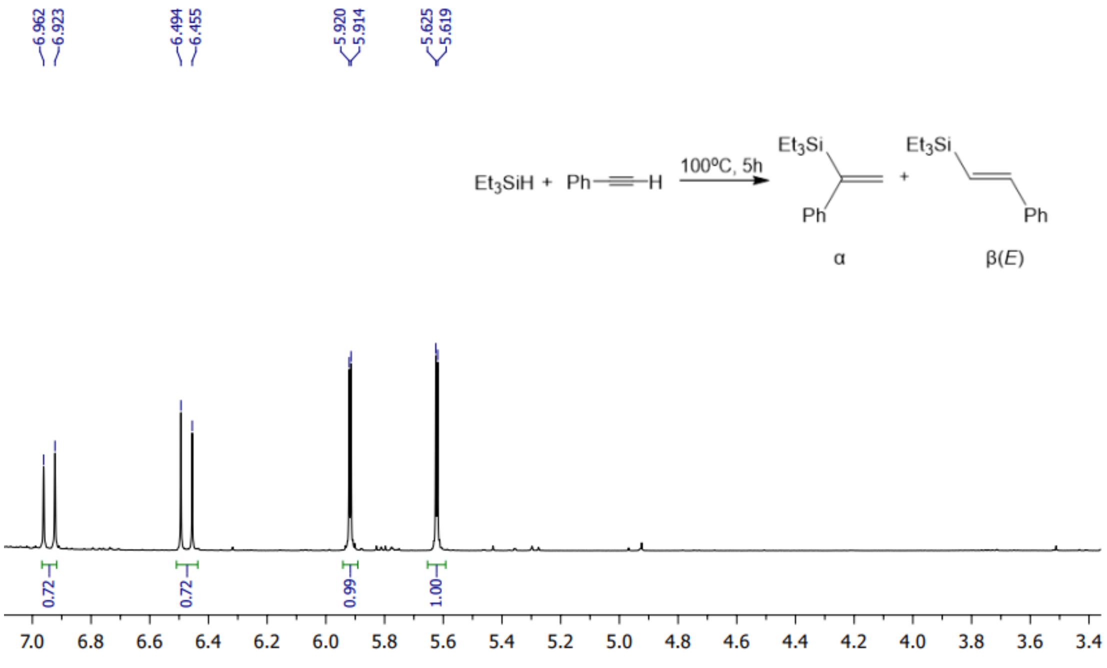

Figure S4. ^{1}H NMR of the product from entry 1

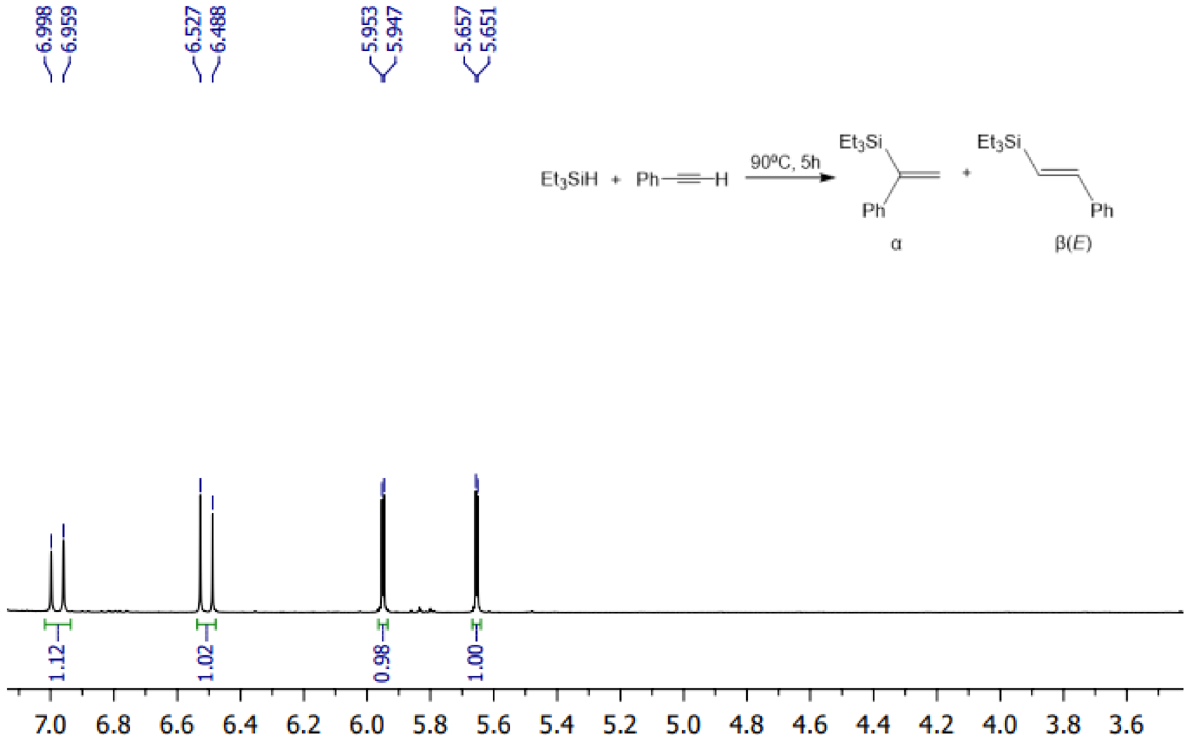

Figure S5. ^{1}H NMR of the product from entry 2

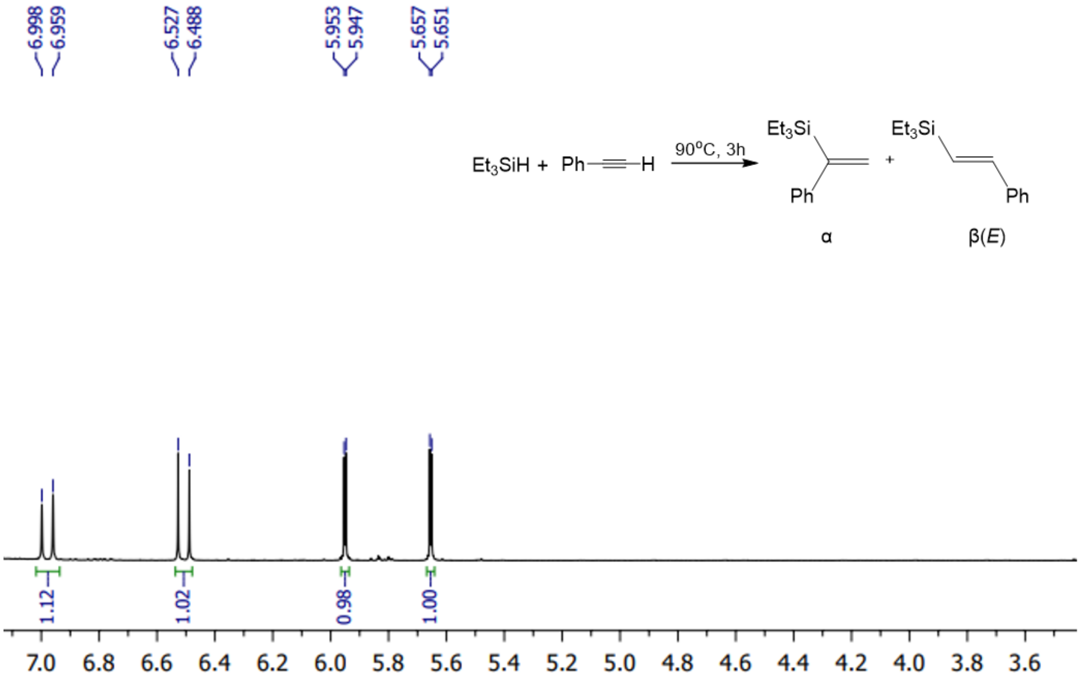

Figure S6. ^{1}H NMR of the product from entry 3

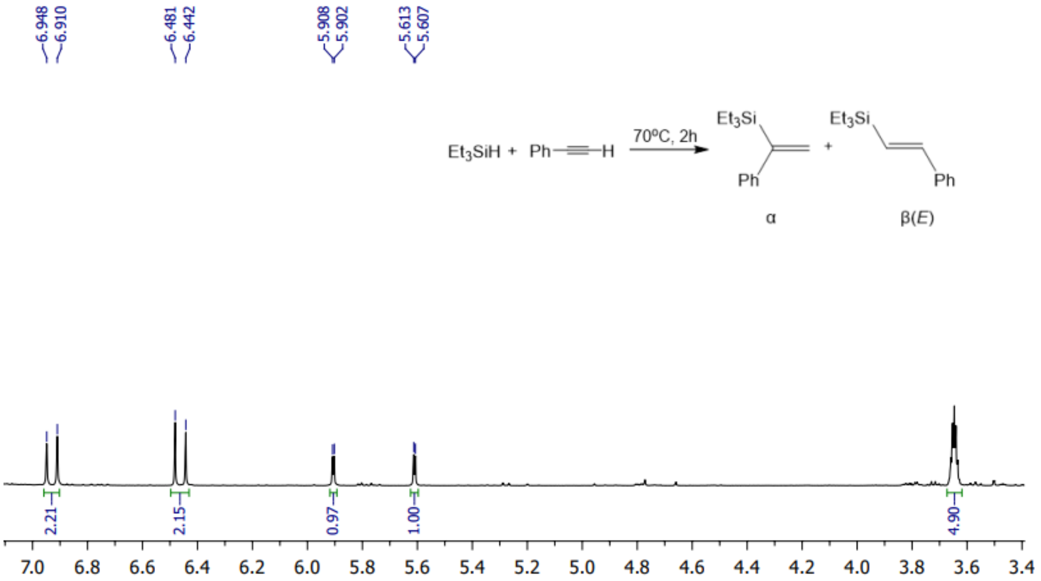

Figure S6. ^{1}H NMR of the product from entry 4
